# Parents' knowledge and predictions about the age of menarche: experimental evidence from Honduras

**DOI:** 10.1186/s13690-023-01030-5

**Published:** 2023-02-10

**Authors:** Michela Accerenzi, Pablo Brañas-Garza, Diego Jorrat

**Affiliations:** 1 Fundación ETEA - Development Institute of Universidad Loyola Andalucía, Córdoba, Spain; 2 Loyola Behavioral Lab, Universidad Loyola Andalucía, Córdoba, Spain

**Keywords:** Age of menarche, Self-report, Guessing, Prediction accuracy

## Abstract

**Background:**

Access to accurate, timely and age-appropriate information about menarche is an essential part of menstrual health. Reliable evidence shows that girls primarily obtain information from their mothers and/or other female family members, therefore, it is important to determine parents’ knowledge and their predictions about other parents’ knowledge of the age of menarche.

**Methods:**

To this end, we performed a pre-registered study with data collected from 360 households in Santa Rosa de Copán, Honduras. We implemented a novel procedure to avoid social desirability bias whereby participants answered two separated questions: *i*) their knowledge about the age of menarche (self-report) and *ii*) to predict or guess the modal response of the other participants regarding the same question (modal guess). Participants were paid according to accuracy. Both questions appeared randomly in the survey.

**Results:**

Recent studies indicate the age of menarche at 12 years old and 56.11% of the sample gave the same response while 62.78% hit the modal value. We estimated the impact of different sociodemographic variables and found only marginal differences. Interestingly, people with formal education and women tend to respond with lower predictions.

**Conclusion:**

Parents’ knowledge about the age of menarche is high in the study area. The study also found that there was no social desirability bias.

**Supplementary Information:**

The online version contains supplementary material available at 10.1186/s13690-023-01030-5.

## Background

Until relatively recently, the topic of menstruation has been overlooked both in international conventions on human rights [5] and in body politics in development. The first attempts to address the issue in an international context originated primarily within the Water, Sanitation, and Hygiene sector in Africa and Asia, and have largely focused on Menstrual Hygiene Management (MHM)^1^ [28].

MHM programs have mainly been implemented in schools and are based on the general assumption that poor girls in low and middle income countries (LMIC) share the same situation: *lack of information* about the menstrual cycle, shame and discomfort during menstruation due to cultural myths and taboos, limited choices about affordable products, insufficient access to private and safe facilities to manage bleeding and menstrual products, and high school dropout rates due to difficulties relating to menstruation [8, 13, 21, 24, 25, 27, 31]. However, evidence to support the efficacy of MHM programs is far from conclusive [1, 2, 4, 10].

A common limitation of such programs is that they often fail to include parents in their activities [4]. However, it is important to acknowledge that parents are hugely influenced by social norms and are responsible for making the decisions on this matter at the household level. Hence, both parents’ knowledge and their social norms play a critical role in the information that adolescents receive and how they behave.

As a consequence, in this paper we focus on *parents’ knowledge and predictions about the age of menarche*. We explore whether parents have accurate knowledge about the age of menarche and whether they are able to predict or guess the distribution of the modal value of other parents’ responses, in other words, the social norm [15, 22]. To overcome possible social desirability bias^2^ in the study [14, 16, 26], we paid participants based on the accuracy of their answers (50 Honduran Lempiras if they hit the right answer).

There are two important aspects to consider in this study. First, access to accurate, timely, and age-appropriate information about menstruation is an essential part of menstrual health [11]. Evidence shows that girls primarily obtain information from their mothers and/or other female family members [7, 18, 29, 32]. Hence, determining whether parents are adequately informed is of paramount importance, given that if they are misinformed they might not only provide girls with incorrect information, but they might also provide it too late, which leaves girls unprepared to face their first cycle [29].

Second, most societies have established social norms about how menstruators and others are expected to behave in a given social situation [9, 17]. General societal misinformation about the age of menarche could indicate that what parents consider “normal” is incorrect, which could lead them to making bad decisions regarding their daughters’ health.

To determine to what extent the general assumptions regarding parents’ knowledge about menstruation are accurate in West Honduras, we conducted a pre-registered field experiment in Santa Rosa de Copán (a region where no MHM or similar programs have ever been implemented). Specifically, our main objective is to explore whether parents have accurate knowledge about the age of menarche; and if they can accurately predict or guess whether others parents also have accurate knowledge about the age of menarche. While the first question captures an individual’s knowledge about the age of menarche, the second measures society’s knowledge about the age of menarche.

The rest of the paper is organized as follows. The next section presents the methods and procedures. Section III describes the sample. Section IV focuses on the results and Section V presents the conclusions.

## Methods and procedures

We ran a lab-in-the-field experiment in Santa Rosa de Copán (Honduras) from May 1–14, 2019. The inclusion criteria for the study was having at least one child between the age of 6 and 9 registered at one of 11 different public schools from different districts (Osorio, El Carmen, Prado Alto, and Santa Teresa). According to the last census (2013),^3^ the town’s population between 6 and 9 years old was 3806. Assuming one child for each household, the needed sample was 350 with a 95% of confidence level and 5% of error. Therefore, we recruited 360 parents to participate in the experiment.

The recruiting process was as follows. Using the socioeconomic status (SES) of the school district, we invited 120 parents per stratum (high, middle, and low) to ensure the sample selection included households from different socioeconomic levels. However, to finish the experiment on schedule, we had to increase the observations and invite 2 and 8 additional parents from the middle and high SES strata, respectively, to complete the 360 observations. Despite this, proportions test suggests that the sample was equally distributed across the three SES categories.

Participants were asked two separated questions: *i*) their knowledge about the age of menarche (self-report *SR*), and *ii*) to predict the modal response of the other participants regarding the same question (modal guess *MG*).^4^ Appendix A shows the original instructions (in Spanish) and B the translation in English. It is important to highlight that SR and MG are not necessarily correlated. While SR captures an individual’s knowledge about the age of menarche, MG measures society’s knowledge about the same subject.

Our design considers both incentives and possible order effects. We used a monetary incentive in the MG task (a monetary award was given if the mode was hit and 0 otherwise) to reduce social desirability bias. Given that the order of the questions may also contribute to bias (see [6], we randomized the question order using *p* = *0.5* to SR → MG and *1-p* to MG → SR. As a result, half of the participants (*n* = *186*) answered SR → MG, and the other half (*n* = *174*) MG → SR (see Appendix A and B).

We also collected participant sociodemographic characteristics to assess possible biases, primarily: sex, education, ethnic group, and socioeconomic status, as well as the composition of the household in terms of girls and boys.

The field experiment was conducted by a Honduran organization, PILARH. Enumerators were trained on the objectives of the study, how to conduct the survey, confidentiality, and informed consent. Before the implementation, the questionnaire’s language was reviewed by PILARH and ETEA Foundation Honduras teams. Then, it was pre-tested in the field with 24 adult participants to ensure that was culturally appropriated and comprehensible. At the same time, the pre-test served to complete the enumerators’ training to avoid any bias induced by their way of introducing the questions. After the survey, PILARH entered the data in an excel spreadsheet. Using Stata, we transformed the categorical or string variables into numerical variables. Only 4 participants chose not to answer their education level, and 1 refused to provide his/her age. That is, we had 355 observations with no missing values for all the variables.

Enumerators used paper-based questionnaires and received a list of households they had to visit, including the type of questionnaire (treatment) they had to implement. Face-to-face interviews were conducted in households and only one experimental subject was interviewed per household (father, mother, or guardian). The random allocation of participants into (order) treatments was made prior to the visit, therefore the enumerators had no influence on the selection.

## Sample and outcome variables

The final sample was 360 participants, 50 were men and 310 women. The respondents from the socioeconomic groups were divided as follows: 31% were from low-income, 34% from middle income, and 35% from high income households. The age of respondents varied from 22 to 78 with the following frequencies: 22–25 (15%), 26–30 (28%), 31–35 (21%), 36–40 (16%), 41–45 (9%), 46–50 (4%), and over 50 (7%). Most respondents over 50 were grandparents.

The respondents cover the entire spectrum of level of education, although most are concentrated in the lower levels: 49% primary education (6 years of schooling) or less, while only 3% held a university degree or higher.

As regards ethnicity: 11% were Chorti, 7% Lenca, almost 8% Maya Chorti, 70% Mestizo, and 4% were from other groups.

In order to assess poverty levels, respondents were asked about access to food in the week previous to the survey: 23% responded that they did not have enough money to feed their children.

Household composition was also determined to assess whether parents with at least one daughter were more informed than those with only sons: 35% of responders had only male children, whilst 65% had at least one female child, but only 21% had at least one daughter who was at least 12 or older (*experience*). Appendix C shows the sample’s sociodemographic data.

The main objective of this study was to determine whether parents had knowledge of the age of menarche and what they believe regarding other parents’ knowledge. The age of menarche varies across countries and time, yet is considered healthy when it happens starts between the ages of 9 and 16. In Honduras, a recent study found that 93.3% of respondents had their first menstruation at 12 [30].^5^

As can be seen below, our sample average is 12.13 with a mode exactly equal to 12. Using the data on the mean age of menarche in Honduras and the modal value of 12 from the sample, we defined the following outcome variables:

- *Self-report: SRHit* (takes the value of 1 if respondents answer 12 and 0 otherwise), *SRUnder* (= 1 if reported age is lower than 12 and 0 otherwise) and *SROver* (= 1 if reported age is higher than 12 and 0 otherwise).
- *Modal guess: MGHit* (takes the value of 1 if respondents guess 12 and 0 otherwise), *MGUnder* (= 1 if respondents guess lower than 12 and 0 otherwise) and *MGOver* (= 1 if respondents guess higher than 12 and 0 otherwise).

Therefore, the first set of items – *SRHit, SRUnder,* and *SROver –* determine whether parents have accurate knowledge while the second set – *MGHit, MGUnder,* and *MGOver –* explores whether they think other parents are also well informed.

## Results

**Fig. 1:**
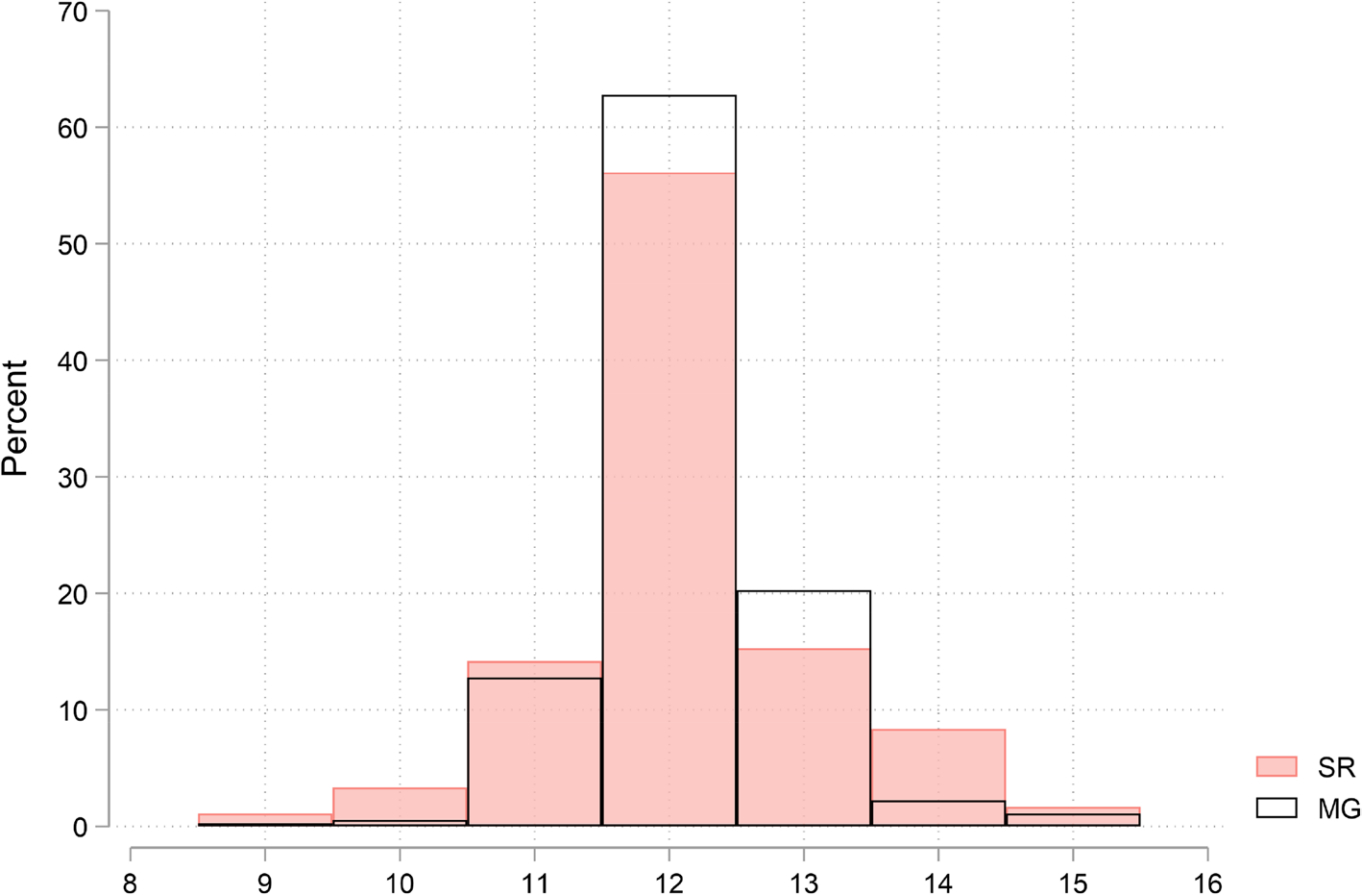
Distribution of self-report responses and modal guesses about the age of menarche

Figure 1 shows the distribution of self-report answers, which highlights that the majority of the sample (56.11%) reported the exact value of the age of menarche, in other words, *SRHit* = *1*. Those who over/under reported are fairly distributed across the range, in fact, *SROver* = 25.28% and *SRUnder* = 18.61%.

Table 1 provides the regression results for the outcome variables. The *order* dummy controls for the order of the questions. The variable *minority group* is equal to 1 if the respondent belongs to an ethnic minority group (Lenca, Chorti, Maya Chorti) and 0 if they are mestizo.

**Table 1:** Regression results for self-report, modal guesses, and informed variables

	(1)	(2)	(3)	(4)	(5)	(6)	(7)	(8)
	*SRHit*	*SROver*	*SRUnder*	*MGHit*	*MGOver*	*MGUnder*	*Informed*	*Misinformed*
*Age*	-0.005*	0.003	0.002	-0.002	0.002	0.001	-0.003	0.003
	(0.003)	(0.003)	(0.002)	(0.003)	(0.003)	(0.002)	(0.003)	(0.003)
*Sufficient income*	-0.014	-0.030	0.043	-0.081	0.073	0.008	-0.028	0.066
	(0.063)	(0.057)	(0.047)	(0.060)	(0.051)	(0.044)	(0.064)	(0.053)
*Education (respondents)*	0.007	-0.018***	0.011**	0.008	-0.012**	0.004	0.005	-0.010*
	(0.006)	(0.006)	(0.005)	(0.006)	(0.005)	(0.005)	(0.006)	(0.006)
*Task order*	0.015	-0.027	0.012	0.047	-0.076*	0.028	0.000	-0.062
	(0.052)	(0.045)	(0.041)	(0.051)	(0.044)	(0.037)	(0.053)	(0.046)
*Experience (at least 1 daughter age* > *12)*	-0.105	0.021	0.084	-0.169**	0.060	0.108**	-0.138**	0.136**
	(0.066)	(0.057)	(0.055)	(0.066)	(0.058)	(0.053)	(0.064)	(0.063)
*Female*	-0.071	-0.074	0.145***	0.053	-0.096	0.043	0.002	0.019
	(0.095)	(0.092)	(0.054)	(0.096)	(0.092)	(0.058)	(0.097)	(0.086)
*Minority*	-0.267*	0.157	0.110	0.039	-0.042	0.003	-0.137	0.091
	(0.142)	(0.144)	(0.106)	(0.141)	(0.132)	(0.093)	(0.141)	(0.131)
*Female and Minority*	0.280*	-0.139	-0.141	-0.056	0.037	0.019	0.136	-0.088
	(0.155)	(0.154)	(0.115)	(0.153)	(0.143)	(0.100)	(0.154)	(0.141)
Constant	0.762***	0.391***	-0.153	0.669***	0.338**	-0.007	0.590***	0.158
	(0.162)	(0.151)	(0.109)	(0.164)	(0.154)	(0.096)	(0.167)	(0.148)
Observations	355	355	355	355	355	355	355	355
R-squared	0.031	0.054	0.035	0.037	0.037	0.025	0.026	0.043

*Note*: Columns 1 to 8 present the OLS estimations of the explanatory variables on the different outcome variables. Robust standard errors in parentheses. Asterisks represent different significance levels: *** *p* < 0.01, ** *p* < 0.05, * *p* < 0.1

Column 1 shows that *age* and belonging to a *minority group* reduce the probability of providing the right answer, however this effect is only marginally significant (*p* = *0*.09 and *p* = *0*.06).^6^ Interestingly, the interaction between *female* and *minority* shows positive but marginal effects (*p* = *0.07*). Column 2 shows that respondents with higher *education* are less likely to overestimate(*p* = *0.001)*, but more likely to underestimate the age of menarche (Column 3, *p* = *0.036*).^7^ In turn, *female* shows a significant and positive coefficient in *SRUnder*, suggesting that women tend to underestimate the age of menarche.^8^ However, the result in Column 3 must be considered with caution since the sample is not balanced by sex since only 14% of participants were men*.* The other variables (*sufficient income, experience, minority*, and *task order*) have no effect on any of the three outcomes.

### Result 1

Most of the sample had accurate knowledge about the age of menarche.

We now focus on the results of the respondents’ predictions or guesses about other respondents’ knowledge as regards the age of menarche. As well as self-report responses, Fig. 1 also shows the distribution of guesses. A significant percentage of the sample, 62.78%, hit the modal value. As in the case of self-reported data, those who over or underestimate are fairly distributed along the range.

In Column 4, Table 1, we estimated the probability of hitting the modal age answered by others. Respondents with *experience* have a lower probability of hitting the modal age (*p* = *0.01)* and are more likely to underestimate the mode (*p* = *0.04)*. The rest of the variables are not significant. Column 5 shows that *education* and *task order* reduce the probability of overestimating the modal age (*p* = *0.03, p* = *0.08*,^9^ respectively*)*. The other control variables have no significant effects.

### Result 2

Most of the sample accurately guessed the modal value of the age of menarche.

Finally, we combined both self-report and guesses for each participant to ascertain an overall measure of their level of information.^10^ Participants were labelled *informed* when *SRHit* = *MGHit* = *1* and *misinformed* when *SRHit* = *MGHit* = *0.* We found that 45.56% of the sample belong to the former category while 26.67% fall into the latter.

Columns 7 and 8 show that parents with *experience* are less likely to be informed while parents with higher *education* have a negative and marginal effect on the probability of being misinformed. Interestingly, the other variables have no impact on the level of information.

### Result 3

A large percentage of the sample had accurate knowledge about the age of menarche and accurately guessed the level of knowledge of the other respondents.

Overall, Results 1–3 show that the parents in our sample were well informed: not only do they have accurate knowledge about the age of menarche, but they also accurately guessed the level of knowledge of the other participants. Table 3 of Appendix D shows the regression results controlling for SES categories and none of the dummy variables (middle and high SES) are significant, suggesting that people are well informed regardless their socioeconomic level. Therefore, results are robust to controlling for different socioeconomics levels.

It should be noted that our results are lower bound. If we considered a more generous definition of Hit, for instance, letting subjects make an error of “ ± 1 year” we would get even better results. In particular, *SRHit* would increase from 56.11% to 85.56%, *MGHit* from 62.78% to 95.83% and *SRHit* = *MGHit* from 45.56% to 83.05%. Therefore, as a rule, it should not be assumed that parents are uninformed.

## Discussion

A common hypothesis behind Menstrual Hygiene Management (MHM) programs is that poor adolescent girls in LMIC do not receive accurate, timely, and age-appropriate information about menstruation. Studies across the world show that adult members of the family, especially mothers and other female members, are one of the primary sources of information for adolescents on reproductive health. However, MHM programs tend to consider that mothers and other family members are not informed [12]. Nevertheless, as far as we know, this is the first research that asks directly to parents if they know the age of menarche.

Using data from 360 households, we determined that knowledge about the age of menarche is high among parents in Santa Rosa de Copán given that 56.11% reported the precise age of menarche that coincides with recent studies (provided by [30]. Moreover, respondents were also able to predict (in a significant 62.78%) the modal response of other participants.

Interestingly, differences in knowledge are poorly explained by sociodemographics. Variables such as *education* and *experience (at least 1 daughter age* > *12),* both with the expected sign, only have marginal effects. These results are in line with Mbugua [20], who found that educated mothers in urban Kenya experience sociocultural and religious inhibitions that hold them back from providing meaningful sex-education, including information about menarche, to their pre-adolescent and adolescent daughters.

More educated people may know that the age of menarche has decreased over time [19, 23] and therefore underestimate the current age of menarche. This could also be the reason they believe that other parents will respond higher values than those responded by themselves. However, this might also relate to prejudices within the local population. It is also important to consider that people might rely more on their own personal experience than on external information, as our results show. Further research is thus needed to understand the correlation between education level, access to information and social norms.

In accordance with Baumann et al. [3], who found that caste/ethnicity was a significant predictor of menstrual knowledge and practices in Nepal, we also found that misinformation is related to *minority ethnic groups,* although only marginally. An unexpected result was that *females* (compared to males) are more likely to underestimate the age of menarche (*p* = *0.001*). Although this result has to be considered with caution because the sample is not gender balanced, a possible explanation is that women rely more on personal experience.

Another interesting result is that no differences were found between the self-reported data and the guesses about the collective modal response. The latter implies that there was no social desirability bias, which suggests that the age of menarche is not such a sensitive topic in Honduras as it is in other cultures. A possible explanation is that the age of menarche might be less problematic than other topics surrounding the menstrual cycle. In fact, another research on the topic in Santa Rosa de Copán found that the menstruation is still surrounded by misinformation, myths and gendered social norms [2]. Therefore, with the information at hand, we can’t reach any conclusion about why adult people have correct information about the age of menarche in a context where misinformation and social norms about the menstrual cycle are prevalent. In order to address these issues, further research is needed.

## Conclusion

This study found that the knowledge about the age of menarche is high among parents in Santa Rosa de Copán. Sociodemographic variables only marginally explain the results and there was no social desirability bias. These results show that one of the common hypotheses behind Menstrual Hygiene Management (MHM) program is not true in the study area. Nevertheless, further research is needed to understand the quality of society’s knowledge about the menstrual cycle as well as to what extent parents transmit this knowledge to their daughters.

### Supplementary Information

**Additional file 1.**

## Data Availability

All of the main data has been included in the results. Additional materials with details may be obtained from the corresponding author. Data in Stata file is also available at the link: https://www.dropbox.com/s/2yzuuqfz0tb1ti7/data_menarche_paper.dta?dl=0.

## Appendix A (Spanish) – Cuestionario sobre menstruaciones y derechos sexuales y reproductivos

### Pregunta auto-informada

I01. ¿A qué edad cree usted que una niña tiene su primera menstruación? ___________ (número > 0).

### Conjetura modal

I02. Hemos preguntado a otras personas de Santa Rosa a qué edad tienen las niñas su primera menstruación. ¿Cuál cree usted que ha sido la edad que han dicho más veces? Piénselo bien porque si acierta, le vamos a pagar 50 lempiras (margen de error ± 1) ______________ (número > 0).

Al final de la encuesta, realizamos el pago si el sujeto había respondido la edad modal de 12 años. También pagamos si la edad respondida era mayor o menor en 1 año.

## Appendix B—Menstruation and Sexual and Reproductive Rights questionnaire

### Self-report question

I01. At what age do you believe girls have their first menstruation? _________ (number > 0).

### Guessing game

I02. We have asked other people in Santa Rosa at what age they believe girls have their first menstruation. With what age do you think people responded most frequently? Think carefully about your answer because if you get it right, we will pay you 50 lempiras (error margin: ± 1 year) __________ (number > 0).

At the end of the survey, we paid respondents if they gave the mode age of 12. We also paid them if the age they gave was higher or lower by 1 year.

## Appendix C

Table

**Table 2:** Summary of the sample's sociodemographic variables

Variables	Frequency	Percent
*Gender*		
Male	50	13.9%
Female	310	86.1%
*Socioeconomic level (SES)*		
Low	110	30.6%
Middle	122	33.9%
High	128	35.6%
*Age (by range)*		
22–25	53	14.7%
26–30	102	28.2%
31–35	76	21.1%
36–40	57	15.8%
41–45	32	8.9%
46–50	14	3.8%
Over 50	27	7.5%
*Education level*		
None	21	5.9%
Incomplete primary education	56	15.7%
Complete primary education	96	27.0%
Incomplete middle school education	28	7.9%
Complete middle school education	29	8.1%
Incomplete secondary education	15	4.2%
Complete secondary education	51	14.3%
Technical/ vocational education	31	8.7%
Incomplete university education	20	5.6%
University education	8	2.3%
Postgraduate education	1	0.3%
*Ethnicity*		
Chorti	39	10.8%
Lenca	27	7.5%
Maya Chorti	28	7.8%
Mestizo	251	69.8%
Garífuna	4	1.1%
Tolupán	3	0.8%
Misquito	3	0.8%
Tawahaka	1	0.3%
Other minority group	4	1.1%
*Access to food the previous week (poverty)*		
Yes	278	77.2%
No	82	22.8%
*Experience*		
At least one daughter 12 or older	77	21.3%

2.

## Appendix D

Table

**Table 3:** Regression results for self-report, modal guesses, and informed variables

	(1)	(2)	(3)	(4)	(5)	(6)	(7)	(8)
	*SRHit*	*SROver*	*SRUnder*	*MGHit*	*MGOver*	*MGUnder*	*Informed*	*Misinformed*
*Age*	-0.004	0.003	0.002	-0.002	0.002	0.001	-0.003	0.003
	(0.003)	(0.003)	(0.002)	(0.003)	(0.003)	(0.002)	(0.003)	(0.003)
*Middle SES*	0.023	-0.035	0.012	0.036	-0.036	0.000	0.059	-0.000
	(0.065)	(0.056)	(0.050)	(0.065)	(0.058)	(0.045)	(0.066)	(0.059)
*High SES*	-0.098	0.077	0.021	0.049	-0.042	-0.007	-0.024	0.024
	(0.068)	(0.061)	(0.056)	(0.067)	(0.058)	(0.051)	(0.068)	(0.062)
*Education (respondents)*	0.011*	-0.022***	0.011*	0.005	-0.010*	0.005	0.007	-0.010
	(0.007)	(0.006)	(0.006)	(0.007)	(0.006)	(0.005)	(0.007)	(0.006)
*Task order*	0.015	-0.026	0.012	0.049	-0.077*	0.028	0.000	-0.063
	(0.052)	(0.045)	(0.041)	(0.051)	(0.045)	(0.037)	(0.053)	(0.046)
*Experience (at least 1 daughter age* > *12)*	-0.105	0.024	0.082	-0.164**	0.057	0.107**	-0.138**	0.132**
	(0.066)	(0.057)	(0.055)	(0.066)	(0.058)	(0.053)	(0.064)	(0.063)
*Female*	-0.056	-0.088	0.144***	0.049	-0.092	0.044	0.010	0.018
	(0.095)	(0.092)	(0.055)	(0.096)	(0.092)	(0.059)	(0.097)	(0.087)
*Minority*	-0.259*	0.145	0.115	0.034	-0.038	0.004	-0.130	0.096
	(0.141)	(0.142)	(0.108)	(0.144)	(0.133)	(0.094)	(0.141)	(0.133)
*Female and Minority*	0.278*	-0.130	-0.149	-0.043	0.026	0.017	0.136	-0.099
	(0.153)	(0.151)	(0.116)	(0.155)	(0.143)	(0.102)	(0.154)	(0.142)
Constant	0.706***	0.417***	-0.123	0.599***	0.405***	-0.003	0.520***	0.215
	(0.161)	(0.152)	(0.109)	(0.162)	(0.154)	(0.094)	(0.165)	(0.149)
Observations	355	355	355	355	355	355	355	355
R-squared	0.041	0.064	0.033	0.034	0.034	0.025	0.030	0.039

*Note*: Columns 1 to 8 present the OLS estimations of the explanatory variables on the different outcome variables. The baseline SES category is low. Robust standard errors in parentheses. Asterisks represent different significance levels: *** *p* < 0.01, ** *p* < 0.05, * *p* < 0.1

^3^.

## Footnotes

1 To understand the magnitude of this phenomenon, see Menstrual Hygiene Day http://menstrualhygieneday.org, Menstrual Health Hub https://mhhub.org/, and Society for Menstrual Cycle Research http://menstruationresearch.org/.

2 While self-reported answers might be truthful or not, incentive compatible mechanisms (paid predictions) are strategy proof. In other words, a subject fares best by being truthful. For example, a country is having elections and there are two political parties: A and B. If a follower of party B is asked who will win, they will say B due to their own preferences/wishes, regardless of the true distribution of votes. In turn, a follower of party A would say A. However, if they are asked to “predict” the results and paid based on their accuracy then their best strategy would be to tell the truth, regardless of their own preferences.

3 The census is available at the Instituto Nacional de Estadísticas de Honduras webpage http://181.115.7.199/binhnd/RpWebEngine.exe/Portal?

4 The ex-ante answer might be any positive (integer) number, although we expected numbers between 8 and 16.

5 Although the study was conducted in the municipality of Choluteca, we consider it to be a good proxy for the situation of Santa Rosa de Copán as a whole.

6 The mean reported by minority groups is 12.10 while the mean reported by mestizos (majority) is 12.14. However, the difference between both groups is not significant (*p* = *0.74*).

7 For each additional year of education, the age of menarche reported decreased by 0.3%.

8 Females report an average age of menarche of 12.10 while males report an average of 12.28. This difference is not significant (*p* = *0.25).*

9 When MG is the second question, the mean is 12.06 and when it is the first, 12.20 (*p* = *0.08*).

10 Both *SR* and *MG* show similar distributions: same mode, similar means (t-test*, p* > *0.40*) and correlation (*rho* = 0.47 (*p* = *0.00)*. In fact, we cannot reject the null hypothesis that both variables present the same distribution (*p* > *0.80*).
